# Some Uses of Chloral Hydrate

**Published:** 1881-12-20

**Authors:** G. A. Collamore

**Affiliations:** Toledo, Ohio


### SOME USES OF CHLORAL HYDRATE.

BY G. A. COLLAMORE, M. D., TOLEDO, OHIO.

Read before the Northwestern Ohio Medical Association at Toledo, June 9, 1881.

Much has been written on the various properties of this drug, both physiological, chemical and therapeutical, and I shall assume that all are familiar with the literature of the subject up to the present time. Very few articles within so limited a period have acquired so extended a reputation in therapeutics. Being primarily mainly employed as a hypnotic, and, perhaps, still maintaining its supremacy in that application, it also has decided claims as an anæsthetic, an anti-emetic, an anti-spasmodic, a febrifuge and antiseptic. This is a large field and one which would require too much time to survey. I shall, therefore, confine myself to the consideration of such employments of it, aside from its chief role of hypnotic, as have been less insisted on by the authorities and which I have at times found especially serviceable, and I shall illustrate my remarks with a few selected cases. In doing this I shall have no theory of its physiological action to support, but shall rather treat the subject empirically, merely desiring to speak well of the bridge which carries me safely over.

As an anti-emetic, chloral acts in suitable cases very promptly and effectually, and suitable cases are those in which the emesis results from derangement of the stomach and bowels, as cholera morbus, cholera, etc., and is not sympathetic, dependent on disease at a distance.

Case I. Mrs. C., aged about 50, had for several days suffered from persistent vomiting and diarrhœa, the ejecta being a thickish, frothy fluid, of a light color, resembling yeast. After the use of opiates, bismuth, etc., with little good effect, chloral was employed in the following manner: A teaspoonful of the solution, containing fifteen grains of the drug, was mixed with fourteen teaspoonfuls of milk, and the patient directed to take one teaspoonful, *i.e.*, one grain of chloral every five minutes until the whole was used. If there was a tendency to vomiting after that, the dose was to be again prepared and taken. It will be seen that in this manner the patient got twelve grains of chloral in the hour. As a result, the vomiting soon ceased and the diarrhœa lessened, the movements becoming darker in color and more consistent. After a day or two all the symptoms disappeared.

This method of administering chloral in minute doses is one that commends itself as introducing the drug in unirritating quantities, and as securing against an overdose. Milk I regard as the best vehicle for its administration per os, as it best covers its pungency.

In certain cases where the vomiting is induced sympathetically, there being no disease of the stomach, chloral does not succeed. Bartholow and others recommend it in the vomiting of pregnancy, but, like all other remedies in that case, it often fails. In cases of vomiting from brain disease, it is also unavailing.

Case II. Miss S., aged 22, had caries of the carpal and metacarpal bones of the left hand. After the disease had existed a year or more, an exploratory operation was made, which resulted in the removal of all the carpal and a part of the metacarpal bones, and the discovery that the ulna was involved nearly to the elbow in the periostitis. In view of the extent of the disease and the debility of the patient, it was deemed best to remove the forearm, which was done at the elbow joint. The wound healed without difficulty. Some two weeks after the stump was entirely healed, she was seized with a severe headache and vomiting. No delirium and but slight fever. Chloral was prescribed in the manner described. The vomiting occasionally ceased, but again returned. The headache persisted. This condition of things continued for nearly a week, when suddenly coma appeared and the patient died in two days. I have given but a sketch of the case, merely to show the inefficiency of chloral to control this species of vomiting.

As an anti-spasmodic, chloral has given me at times great satisfaction, particularly in the convulsions of children.

Case III. I was called to see a child, 2½ years old, who was called nervous and had had convulsions some months previously. The child, a girl, was thought to have symptoms of convulsions, and I had been in the room but a few minutes when it was actually seized with them. Not having any chloral with me, I despatched a messenger to the nearest drug store for some, and as I had heard that the nitrite of amyl had been used as an anti-spasmodic, and happening to have some at the time, I begun to give it by inhalation, partly to try its effects and partly to be doing something pending the arrival of the chloral. I do not now remember how much of the amyl I gave in all, but the doses I did give, gradually increased from two or three to ten or twelve drops, had not the slightest apparent effect in controlling the convulsions. I must have given more than thirty drops in this way during the half hour I was waiting for the chloral. The messenger finally got back with the report that the druggist had no chloral, but would send for some. Not being inclined to wait an indefinite time, I went to my office, got the medicine and returned. There had as yet been no remittance in the spasms. Finally, forty-five minutes from their commencement, I administered, per rectum, about eight grains of chloral, and in less than three minutes the convulsions had ceased, and there was no return. Subsequent events showed that the cause of the trouble was malarial, and the disease was promptly subdued by quinine.

The use of chloral by injection in such cases is, in my opinion, preferable, as it is easily administered, very rapidly absorbed and produces its effect even more promptly, as it appears to me, than when given by the mouth. It must also be remembered that no larger dose is required or will be borne by the rectum than the stomach.

As an anti-spasmodic I have also derived much benefit from chloral in the after-pains of multipara. Practically, but a small proportion of my patients suffer from this cause, which I attribute, whether correctly or not I cannot say, to great carefulness in removing the placenta, so that no strings or shreds of membrane are left behind. When after-pains do oecur, however, chloral relieves them readily, and it is rarely necessary to give but a few 5 to 8 grain doses. Here I prefer the use of milk as a vehicle.

There is but one other application of chloral to which I shall invite your attention. There is a species of sore throat, characterized by moderate swelling of the tonsils and adjacent mucous membranes, pain in deglutition and a peculiar cherry-red or purplish-red hue of the tonsils and pharynx. On the tonsils appear spots of whitish or yellowish white color, the size of a grain of corn or less. These are composed of the aggregated secretions of the tonsillar glands, and are readily detachable, leaving the mucous surface unabraded. There is, moreover, a moderate, sometimes high grade of fever and decided prostration of the system. The disease is properly a follicular tonsillitis, though the inflammation is not confined to tonsillar surfaces, but affects the palatine and pharyngeal mucous membrane also, and is liable to be mistaken for and called diphtheria, from which a little care in observation will differentiate it.

It is in these cases, combined with suitable, systematic remedies, that chloral acts in a kindly manner as a local application, either as a gargle, a grain or two to the ounce of water, frequently used, or in a stronger solution applied with a camel's hair brush or a swab. I usually direct a small quantity of the gargle to be swallowed after each gargling, in order to apply it to the lower pharynx. I have employed chloral in this way many times, and think my patients have derived benefit from it.—*Detroit Lancet.*

